# Surveying 80 Years of Psychodrama Research: A Scientometric Review

**DOI:** 10.3389/fpsyt.2021.780542

**Published:** 2021-11-15

**Authors:** Mengyu Lim, Alessandro Carollo, S. H. Annabel Chen, Gianluca Esposito

**Affiliations:** ^1^Psychology Program, School of Social Sciences, Nanyang Technological University, Singapore, Singapore; ^2^Department of Psychology and Cognitive Science, University of Trento, Rovereto, Italy; ^3^Centre for Research and Development in Learning (CRADLE), Nanyang Technological University, Singapore, Singapore; ^4^Lee Kong Chian School of Medicine, Nanyang Technological University, Singapore, Singapore; ^5^Office of Educational Research, National Institute of Education, Singapore, Singapore

**Keywords:** psychodrama, Moreno, scientometry, systematic review, document co-citation, psychotherapy, keyword analysis

## Abstract

Almost a century after Jacob Levy Moreno pioneered the group practice of psychodrama, research in this area has flourished to include different sub-fields of study and psychodramatic intervention for various psychological conditions. By making use of scientometric analysis, particularly document citation analysis and keyword analysis, this study maps out dominant research domains in psychodrama since its inception. From these findings, projections of future research trends and an evaluation of psychodrama research are discussed. Generally, there has been an increased adoption of technology to facilitate psychodrama practice, along with an increasing integration of psychodramatic principles with other psychotherapies. To improve research in this area, this paper recommends greater transparency in the reporting of materials, processes and data used in publications. Finally, we encourage embracing new technological methods such as neuroimaging to provide greater insight into mechanisms of change in psychodrama. The field of psychodrama remains full of potential and innovations to be developed.

## Introduction

Developed almost a century ago by Romanian-born psychiatrist Moreno (1), psychodrama is a form of psychotherapy that utilizes elements of theater, role-play and group dynamics (2). Psychodrama, along with the larger methodological study of interpersonal connections known as sociometry (3), was formally conceptualized by Moreno at the turn of the 1920s in the aftermath of World War I (4, 5). At its inception, psychodrama was the first instance of group psychotherapy, built upon the ideal of an “action method” (4) that allowed participants to act out their problems rather than merely talking about them in traditional “talk therapy” that was popularized by Sigmund Freud's psychoanalysis in the nineteenth century (6). Despite the global backdrop of war and conflict of that time, the nature of psychodrama in psychotherapy has always been that of a positive, humanistic perspective (5). Psychodrama was innovative for its time, especially as the “acting out” of maladaptive tendencies and neuroses was thought to be harmful to the patients in traditional psychotherapy (4).

There are two factors that Moreno proposed that distinguish psychodrama from other forms of psychotherapy: spontaneity and creativity (2). Spontaneity refers to the ability to express oneself freely and adequately in novel situations and catalyzes the process of creativity, creating art and “cultural conserves” in the process (2). According to Gershoni et al. (7), cultural conserves are formed from the interaction between spontaneity and creativity, serving as the basis of creativity and shaping creative expression within the individual. By nurturing spontaneity and creativity in the individual during group psychodrama, Moreno argues that a surplus reality can be created: a version of reality that is the externalization of an individual's subjective reality (2, 4). The dramatic re-creation of the subjective reality enables the individual in question to role-play other types of behavioral patterns, thereby resulting in therapeutic change. There are several different techniques of role-play employed in psychodrama, the most prominent of which being role reversal where an individual (A) takes the role of another (B) and vice versa (1, 8). Role reversal has been seen as the most effective technique in psychodrama (9, 10). Holmes et al. (11) propose three phases in achieving role reversal: firstly, the cognitive process of empathic role taking; secondly, the behavioral process of reproducing the role in one's actions; thirdly, the social cognitive process of role feedback where role-players' subsequent responses are based on a bi-directional, interactive perception of each other. Other role-playing techniques include mirroring, where A takes the role of B while B observes, and doubling, where A takes the role of B alongside B (12). Finally, it has been suggested that other roles may be replaced by inanimate objects, such as in the “empty chair” technique (13).

Since the peak of the psychodrama movement in the 1950s, multiple studies have demonstrated the effectiveness of psychodrama and role-playing in producing therapeutic outcomes (14–16) by changing role expectations (17), as well as through improving interpersonal skills (18) and role expansion (4, 19). Currently, psychodrama has since been adapted and practiced in more than a hundred countries across the globe (5). The importance of psychodrama to this day cannot be understated, as its inherent focus on interpersonal relationships is more relevant than ever in today's age of social isolation, driven in part by technological advancement (20) and the COVID-19 pandemic in recent years (21). Given the close relationship between psychodrama and contemporary humanistic approaches in psychotherapy (22, 23), as well as the increasing prominence of research on attachment and interpersonal phenomena (24), it can be seen that psychodrama has massive potential in modern psychotherapy as well as interdisciplinary studies of human relationships and interactions. To date, however, few studies have investigated the effectiveness of psychodrama in relation to the two tenets: spontaneity and creativity (25). Other studies have also made use of psychodramatic role-play techniques as a means of measuring role behaviors as a dependent variable, but such use of psychodrama remains unsubstantiated by validated research (26, 27).

The above identified research gaps notwithstanding, the long history of psychodrama warrants a broad overview of the progress made in this field. Some existing literature has attempted to review psychodramatic research. For example, a recent study by Orkibi and Feniger-Schaal (25) is an integrative systematic review on the state of psychodramatic intervention over a decade, involving systematic searches across four databases and a hand search across both quantitative and qualitative studies. While illuminating and providing important insights to modern psychodramatic practices, the paper was not able to survey the history of psychodrama and research trends over its many decades. Recent meta-analyses such as López-González et al. (28) and Wang et al. (29) are also limited in terms of their focus on only controlled clinical trials and the application of psychodrama within the Chinese culture, respectively, without consideration of other methodologies or theoretical papers and a broader cultural context. On the other hand, older systematic reviews have similarly only captured approximately three decades' worth of psychodramatic research (14, 30, 31). Additionally, the meta-analysis performed by Kipper and Ritchie (14) was lacking in details that assured replicability and allowed for contextual understanding, such as search terms and the names of databases. Taken together, while such reviews are able to evaluate the state of psychodrama research, they were not able to reveal landmark studies as well as key publications that have shaped the field of research and influenced the development of new research trends in the whole of the psychodrama research field. Therefore, scientometric methods are adopted in this paper in an attempt to survey the major research trends in psychodramatic research since its inception, taking a whole-of-domain approach with no restrictions in the time period, geographical area or type of publication.

Scientometry has surfaced as a quantitative methodology to evaluate scientific production in a specific field of study (32), and has been used in the scientific community to examine research trends from the evolution of psychological constructs [e.g., (33)], advances in research methods and techniques [e.g., (34)], as well as content advancement in biology, medicine, physics and many more [e.g., (34–36)]. By using quantitative data such as citation metrics, scientometry offers an objective view of widely used citation pathways within specific domains of study, therefore informing the development of research and research impact of the literature (37). Additionally, scientometric reviews are more useful in capturing more sources than manual reviews (38), particularly as the aim of the present study is to obtain a broad overview of research trends in psychodrama.

At the end of nearly a century of research on psychodrama pioneered by Moreno, this paper aims to use scientometric methods to 1) survey major research trends in this field since its inception, 2) provide insight to future potential directions of research in this area, and 3) evaluate research methods pertaining to psychodrama in current prominent studies to date.

## Methodology

The dataset of publications used for this study was obtained from Scopus, an abstract and citation database run by Elsevier. Only one database was selected in alignment with the recommendation by Chen (39). This is because of several reasons, including 1) the differences in bibliography indexing between databases and 2) the high likelihood of duplicate entries between databases. The bibliographic search was conducted on 10 August 2021. Bibliographic search was conducted with the string of keywords: “TITLE-ABS-KEY (psychodrama^*^),” therefore selecting articles that contain psychodrama and its derivatives in their title, abstract, or keywords. Language was not restricted in this study, as the aim was to include as many relevant papers as possible. Additionally, it was revealed that many studies of psychodrama interventions were conducted in countries where the main language is not English (25). More specifically, by noting the non-English articles excluded in Orkibi and Feniger-Schaal (25), the countries' main language ranges from Persian, Croatian, Turkish, Polish, Portuguese, German, and French. For example, as will be seen later in section 4.1, the article with the largest coverage in the largest cluster originates from Paris, France (40). The final dataset generated by Scopus consisted of 2056 records published between 1943 (precise date unknown) and 21 July 2021. This range of dates was not artificially restricted by the authors, but depended on the earliest and latest available documents indexed by Scopus at the time of the search. CiteSpace software (version 5.8.R1) was used for scientometric analysis. When importing the publication dataset, a total of 2048 records were converted successfully into the Web of Science format used by Citespace, and with them 28,848 out of 30,983 references (93% success conversion rate). According to Chen (41), typical data loss due to irregularity in the original cited references ranges from 1 to 5%. As this dataset had 7% data loss in the references, it may be concluded that psychodrama literature tended to contain more irregular references than an average field of scientific literature. After the initial screening conducted by Scopus based on the keywords, the dataset was not further examined for relevance to psychodrama. This is so that the dataset will not be biased in a systematic way upon manual removal of articles deemed to be irrelevant, similar to the practice in Gaggero et al. (33), Aryadoust and Tan Hah (42), and Lim and Aryadoust (43). Additionally, Chen (39) recommends to defer the screening for relevance to the analysis stage, for the similar reason that ambiguous terms may or may not be relevant to the search query. In fact, as later discussed in section 4.1, the retaining of articles in the original dataset may inform researchers of existing disagreements on how the field of research is constructed and defined.

Document Co-Citation Analysis (DCA) on CiteSpace was conducted to uncover main trends in psychodrama literature. DCA is an analysis of the frequency of co-citation of two or more documents or articles (44), and is based on the hypothesis that high frequencies of co-citations reflect prominent trends and common clusters of research within the examined field of literature (45). Therefore, by operating on this hypothesis, DCA visualization on CiteSpace generates a network of documents that are frequently co-cited, as well as the documents that cite them.

Several DCA networks had to be generated and compared to create a visually balanced network. These networks were generated using three distinct node selection criteria: g-index, Top N and Top N% [as in Carollo et al. (35)]. G-index was initially designed to improve upon the older h-index by taking more heavily into account the citation metrics of an author's most cited publications (46, 47). In Chen (41)'s words, the g-index indicates the “largest number that equals the average number of citations of the most highly cited g publications”. On the other hand, Top N and Top N% are criteria that select the top N or N% cited documents within a time slice as network nodes (48). For the purposes of this study, the time slice was always maintained at 1 year per slice. In this study, g-index with the scaling factor k set at 25, 30, 50, 100, 150 and 200, Top N with N at 25, 30, and 50, and Top N% with N at 1, 5, and 10 were tested. The overall effects of these settings on the generated networks' structural metrics and number of nodes and clusters identified were evaluated by the authors for the final decision on the node selection criteria for DCA analysis.

After the comparison of selection criteria, it emerged that there were a very limited number of links between nodes that were spaced temporally further apart, resulting in clusters that were restricted to the span of a few years (e.g., only clusters from 2000 to 2003 were identified in a sample that ranged from 1943 to 2021). Therefore, the “Look back years” parameter was modified at this stage to a value of –1, implying that all the references cited in a citing paper were considered in the construction of the network, independently from their temporal distance from the source paper. At the same time, the “Maximum links per node” parameter was also set as unlimited, thus allowing CiteSpace to explore all possible links between nodes. Eventually, the best criteria determined by the authors was g-index with scaling factor k at 100 with unlimited lookback years. Therefore, this set of criteria was used to generate the final DCA network of documents.

A preliminary visual analysis of the resulting network revealed that several nodes represented the same article due to irregularities in referencing style. Therefore, 4 nodes (2 each representing the same 2 publications) were merged manually using the CiteSpace alias function, with the primary node designated as the node belonging to the earlier dated cluster.

**Figure 1:**
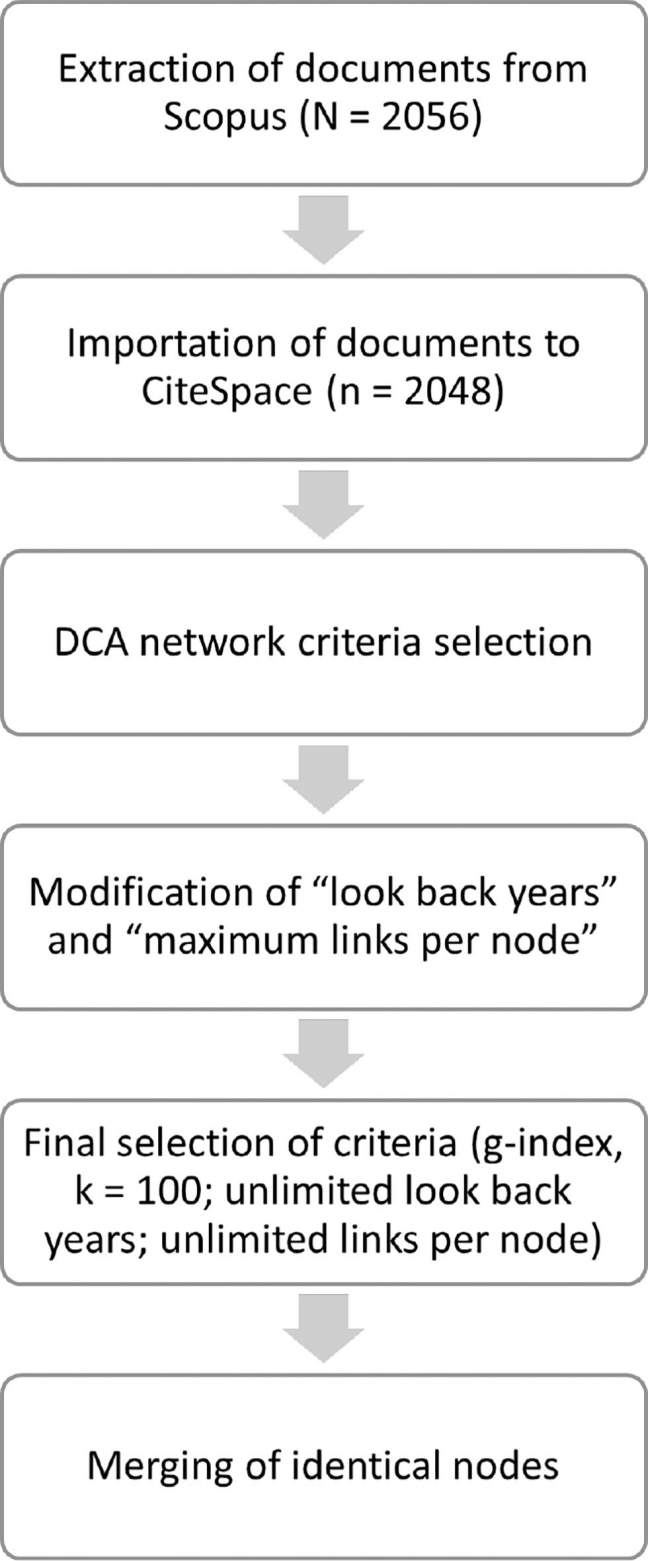
Flowchart of study procedure.

The process of obtaining the final dataset and network visualization is represented in Figure 1.

CiteSpace-generated networks and resulting nodes can be evaluated by using two types of parameters: structural and temporal metrics. Structural metrics include modularity Q, silhouette score and betweenness centrality. Modularity Q has values ranging from 0 to 1, indicating the degree to which the network can be divided into distinct groups of nodes, also called modules or clusters (49). High modularity Q values imply a network with good structure (45). Secondly, silhouette score measures inner consistency (i.e., cohesion and separation) of the clusters (50). Values of silhouette score can range from -1 to 1, with higher values representing a cluster's high separation from other clusters as well as internal consistency within the cluster (51). Lastly, betweenness centrality indicates the degree to which a node functions as a connection between an arbitrary pair of nodes in the network (48, 52). Its values range from 0 to 1, where the highest values are typically obtained by groundbreaking and revolutionary works in the scientific literature (42). The second type of parameters is known as temporal metrics. They include citation burstness and sigma. Calculated with Kleinberg (53)'s algorithm, citation burstness measures the sudden increase in citations a document received within a given period of time (39). Next, the sigma metric is calculated by (centrality+1)^burstness^, and provides quantitative insight into the document's novelty and its influence on the overall network (54). Influential publications have higher citation burstness and sigma. In this study, structural metrics, specifically modularity Q and silhouette score, were used to evaluate overall structural configuration of the network and clusters. Additionally, properties of single nodes were examined using citation burstness.

To provide a complementary perspective to the metrics provided by DCA analysis, a brief keyword analysis was also conducted to understand the common topics, the relationships between them, and trends within the field of psychodrama. In the keyword analysis, the same parameters were used (i.e., g-index with scaling factor k at 100 and unlimited lookback years). Prominent keywords with significant burstness are subsequently reported.

## Results

### DCA Analysis

The final DCA network visualization comprised 5,025 nodes and 15,327 links, where each node represents either a document that has been cited or a document that has cited references. Thus, on average, each node showed 3.05 links with other nodes in the network. A modularity Q score of 0.9794 suggests that the network is highly divisible into distinct clusters. An average silhouette score of 0.9796 suggests that on average, each cluster is highly internally consistent. Taken together, the DCA network yielded a good structure with clusters of research within the psychodrama literature that are distinct from each other, yet are consistent within themselves.

**Figure 2:**
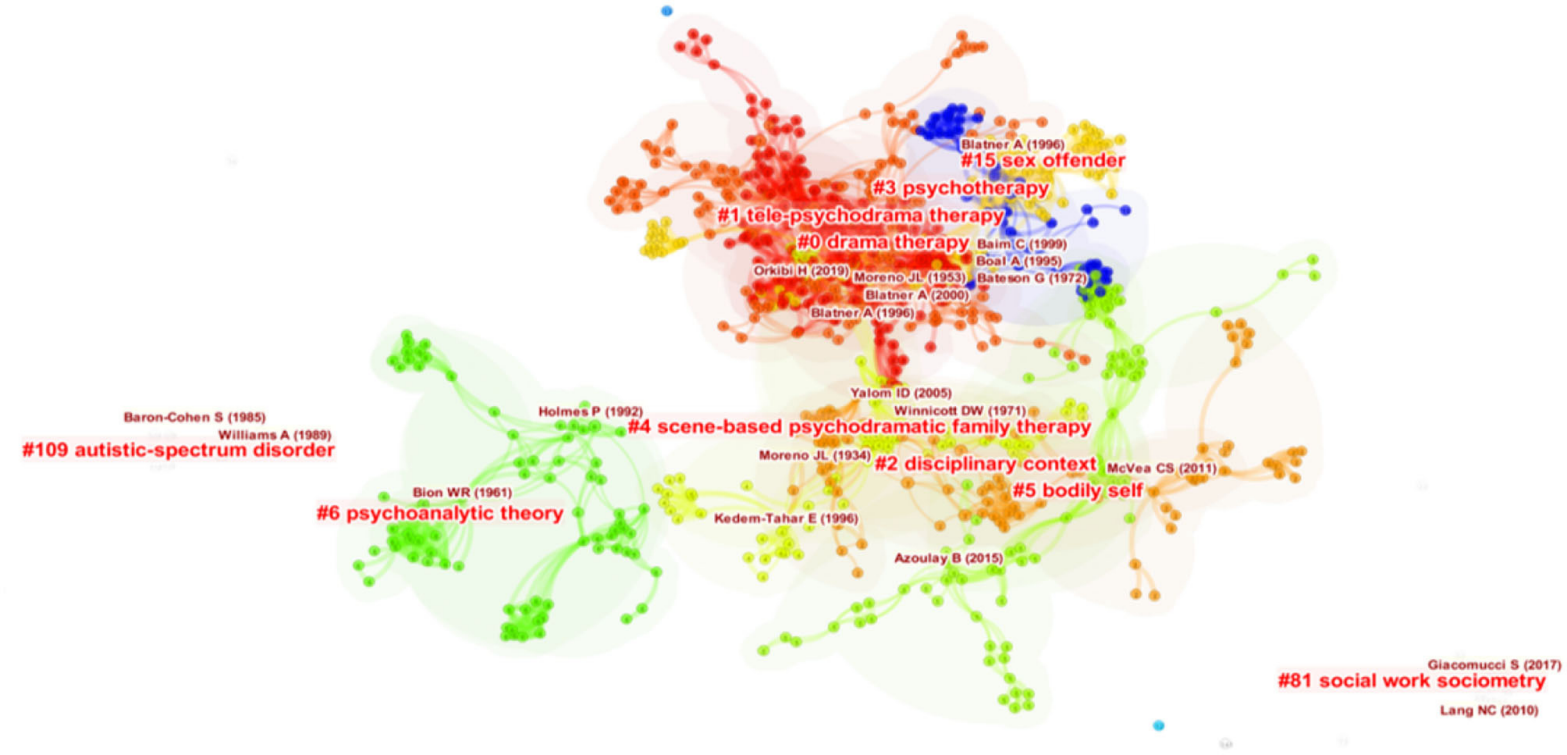
Final Visualization of DCA Network. Smaller clusters such as #38 “User-Friendliness”, as they are located further from the main network, are not included in this visualization.

Thirteen major clusters were identified within the network (see details in Figure 2 and Table 1), the five largest of which will be further discussed in the next section. The five largest clusters were chosen for further examination as they were identified by CiteSpace's Narrative Summary function. This approach of partial reporting has been adapted by numerous other works (35, 55–57), and is particularly suitable for large numbers of clusters [e.g., (58)]. As seen in Figure 2, different colors correspond to different clusters, with a progression from warmer colors (e.g., red) to cooler colors (e.g., blue) as the clusters decrease in size. Considering the average years of publication, major clusters began appearing around the 1980s, and have persisted to more recently. Generally, as indicated by the high silhouette values, all clusters are highly internally homogeneous. As for the size of the clusters, cluster #0 is the largest within the network, consisting of 192 documents with a silhouette score of 0.966, thereby also the least internally homogeneous of all major clusters. Clusters are automatically labeled by the log-likelihood ratio (LLR) algorithm of CiteSpace. Previous research shows that LLR provides the most accurate labeling of clusters among other available methods in CiteSpace in terms of its coverage of documents within the cluster and its uniqueness across clusters (33, 48), although they may still be imprecise when compared to manual cluster labeling (34). Therefore, while the LLR labels are retained in the subsequent discussion of the clusters, an alternative label is suggested where the LLR label may be deemed inadequate by a closer look at the contributing articles of the cluster.

**Table 1:** Metrics of major clusters identified by document co-citation analysis.

**Cluster ID**	**Size**	**Silhouette**	**Mean year**	**LLR label**
0	192	0.966	1990	Drama therapy
1	134	0.973	2003	Tele-psychodrama therapy
2	95	0.976	1984	Disciplinary context
3	92	1	1982	Psychotherapy
4	89	0.984	1993	Scene-based psychodramatic family therapy
5	85	0.984	1997	Bodily self
6	80	0.97	1979	Psychoanalytic theory
15	48	0.994	1992	Sex offender
38	22	0.995	2010	User-friendliness
56	20	1	1980	Reenactment
81	15	0.999	2015	Social work sociometry
109	12	0.996	1993	Autistic-spectrum disorder
116	10	1	1986	Eclectic psychodrama

Two documents showed a citation burst in the network, which gives a measure of the papers' relevance to the psychodrama landscape (see details in Table 2). Specifically, from cluster #1, Moreno (1)'s 2nd edition book titled “Who shall survive? Foundations of sociometry, group psychotherapy and socio-drama” has the strongest citation burst of 12.4349 lasting from 2008 to 2021, an entire half century after its year of publication. The second document with a significant citation burst is the 4th edition of Blatner (59)'s book titled “Foundations of Psychodrama: History, theory and practice.” This document belonged to cluster #0, and has a citation burst of 11.228 lasting from 2013 to 2021.

**Table 2:** Documents with high citation burstness generated by document Co-citation analysis.

**Reference**	**Strength of burstness**	**Burst start**	**Burst end**	**Duration**
Moreno (1)	12.4349	2008	2021	14
Blatner (59)	11.338	2013	2021	9

### Keyword Analysis

The final keyword analysis comprised a network of 2830 nodes and 27106 links, where each node represents a keyword. Thus, on average, each node showed 9.58 links with other nodes in the network. This network showed a modularity Q score of 0.4216 and an average silhouette score of 0.745. 151 keywords showed a citation burst in the network. The top ten keywords with the strongest burst are: therapy (burst = 85.0283), central nervous system (burst = 73.4538), role playing (burst = 69.3624), psychological aspect (burst = 46.7224), article (burst = 42.8839), methodology (burst = 41.0485), english abstract (28.048), priority journal (burst = 27.2888), teaching (burst = 25.4252), education (burst = 25.3455). The top ten keywords with the earliest burst beginnings are article (beginning = 1946), psychotherapy (beginning = 1946), neurotic disorder (beginning = 1946), neurosis (beginning = 1946), hospital (beginning = 1948), mental hospital (beginning = 1948), personality test (beginning = 1951), projective technique (beginning = 1951), group therapy (beginning = 1951), mlown (beginning = 1954). The top ten keywords with the latest burst beginnings are review (beginning = 2019), follow up (beginning = 2019), adult (beginning = 2018), pretest posttest design (beginning = 2018), interview (beginning = 2017), student (beginning = 2017), human experiment (beginning = 2017), aged (beginning = 2015), cognitive behavioral therapy (beginning = 2015), mental health (beginning = 2014). The top ten keywords with the longest duration of bursts are clinical article (duration = 35 years), hospital (duration = 32 years), mental hospital (duration = 32 years), art therapy (duration = 28 years), transference (duration = 27 years), article (duration = 26 years), neurotic disorder (duration = 26 years), neurosis (duration = 26 years), adaptive behavior (duration = 26 years), psychotherapy (duration = 25 years). As the number of significant keywords are large (n = 151), partial findings are reported in Table 3, representing only the top-25 keywords with the highest citation burstness and bottom-10 keywords with the lowest citation burstness, in accordance with Gaggero et al. (33).

**Table 3:** Keywords with significant citation burstness generated by keyword analysis.

**Keyword**	**Strength of burstness**	**Burst start**	**Burst end**
Therapy	85.0283	1973	1987
Central nervous system	73.4538	1978	1987
Role playing	69.3624	1975	1991
Psychological aspect	46.7224	1981	1992
Article	42.8839	1946	1971
Methodology	41.0485	1977	1992
English abstract	28.048	1974	1985
Priority journal	27.2888	2008	2021
Teaching	25.4252	1977	1992
Education	25.3455	1980	1990
Psychology	22.6863	2013	2021
Nursing	22.6532	1979	1995
Procedure	22.0076	2013	2021
Personality test	21.8719	1951	1972
Psychotherapy	20.7545	1946	1970
Controlled study	20.7162	1999	2021
Projective technique	19.8907	1951	1971
Cognitive therapy	18.683	2001	2015
Interpersonal relation	18.4788	1964	1974
Nursing education	17.5613	1977	1990
Major clinical study	15.8708	1973	1977
Attitude	15.6419	1966	1974
Creativity	14.2192	2005	2019
Group therapy	14.0741	1951	1967
Human experiment	13.7474	2017	2021
Motivation	4.6253	1971	1973
Anxiety disorder	4.6232	2003	2021
Caregiver	4.5882	2005	2021
Dementia	4.5753	2012	2018
Group	4.5001	2006	2016
Eating disorder	4.4851	2007	2021
Time factor	4.4673	1969	1975
Assertiveness	4.466	1979	1984
Borderline state	4.4651	2001	2006
Psychotherapist	4.4407	2000	2021

*Only the top-25 highest citation burstness, and bottom-10 lowest citation burstness are reported*.

## Discussion

Based on the DCA network, the five largest clusters will now be discussed in greater detail. Labels of these clusters are generated using Citespace's LLR algorithm. Citing papers contributing to the cluster are reported in terms of their global citing score (GCS; total number of citations of the paper as reported by Scopus) and coverage (number of references in the cluster that are cited by the paper). A third metric, local citing score (LCS; total number of citations of the paper within the dataset of this study), was not included as the citing papers in the following five largest clusters all have a LCS of 0.

### Cluster #0: Drama Therapy

The largest cluster was named “drama therapy” and consisted of 192 references. The citing papers belonging to this cluster are represented in Table 4. As can be seen in Table 4, the most popular avenue of publication for articles belonging to this cluster is Arts in Psychotherapy (Impact Factor 1.404), an international peer-reviewed journal on mental health and creative arts therapy. This finding is also true for the later Cluster #1.

**Table 4:** Citing papers belonging to cluster #0.

**Reference**	**GCS**	**Coverage**
Clit (40)	1	39
Blatner (60)	9	29
Kedem-Tahar and Felix-Kellermann (61)	22	26
Hamamci (62)	41	12
Azoulay and Orkibi (63)	23	10
Molina et al. (64)	8	10
Haberstroh (65)	3	8
Hagedorn and Hirshhorn (66)	8	8
Verhofstadt-Denève (67)	10	7
Blatner (68)	0	7
Kähönen et al. (69)	40	7
Michel and Andacht (70)	0	6
Snow (71)	8	6
Westwood et al. (72)	15	6
Holmes et al. (11)	3	6
Lamiani et al. (73)	2	6
Grinberg et al. (74)	8	6
Olesen et al. (75)	0	5

A survey of the citing papers in this cluster reveals that research in this cluster focuses heavily on the manualization and implementation of psychodrama in clinical settings (e.g., (11, 60, 63, 68)), as well as theoretical discussions of psychodrama in relation to other psychotherapeutic constructs (e.g., (67, 70)). Most papers in this cluster made use of qualitative clinical reports and interviews, although a minority used quantitative analysis and clinical vignettes. The earlier papers of this cluster (e.g., (61, 71)) are primarily interested in the definition of psychodrama and its characteristics, in order to fulfill a clinical niche that is distinct from drama therapy. According to Kedem-Tahar and Felix-Kellermann (61), while psychodrama focuses on therapeutic self-awareness, drama therapy prioritizes aesthetic expression, thereby also producing different outcomes. According to them, psychodrama has its roots in Moreno's philosophy, while roots of drama therapy are decidedly murkier. On the other hand, drama therapist (71) refutes the distinction between the origins of psychodrama and drama therapy, arguing instead that theater and drama have roots in human instinct that pre-date formalization. In contemporary literature, while psychodrama refers to a more specific practice, both psychodrama and drama therapy are now recognized to have therapeutic potential, even if drama therapy belongs to a separate family of creative arts therapy including music, dance, and art therapy. Principles of psychodrama have also become flexible enough to be incorporated into drama therapy and other psychotherapeutic modalities (60). The later dated manuals of psychodrama written by Blatner (59) and Karp et al. (2) then form the basis of the psychodrama practice to this day. Taken together, the proposed label for the cluster may be “Formalization of Psychodrama.”

### Cluster #1: Telepsychodrama Therapy

The second largest cluster was named “telepsychodrama therapy” and consisted of 134 references. The citing papers belonging to this cluster are represented in Table 5. It can be seen that most citing papers of this cluster are fairly recent, with most published within the last 5 years of this study. The label of this cluster originated from Biancalani et al. (76)'s work on adapting psychodrama to an online format during the COVID-19 pandemic, but is also more broadly indicative of research advances in psychodrama in recent years, such as application to themes such as sexuality and gender (82, 94), abuse and trauma (81, 84), in addition to various physical and mental health conditions (77, 80, 93). Furthermore, there is also an increasing focus on preventive work (e.g., (78, 79, 85)), revealing the broader movement of psychological interventions toward a more positivist and humanistic approach. There has also been a trend of integrating elements of psychodrama, or even more loosely, role-playing and theater elements, with other forms of psychotherapy. Unfortunately, while there are indications of an evolution of psychodrama and applications toward broader fields of research, psychodrama intervention techniques seem to have remained fairly unchanged, save for the online adaptation in Biancalani et al. (76)'s paper. Finally, this cluster also showed an increasing focus toward rigorous research and an increased variation in research methodologies, as evidenced by Orkibi and Feniger-Schaal (25)'s integrative review, López-González et al. (28)'s systematic review, and Wang et al. (29)'s meta-analysis in this cluster, among others (e.g., (77, 87)). An interesting opinion on research methodology comes from Yaniv (86)'s paper on spontaneity in psychodrama and its relationship with current neurocognitive theories. Taken together, a more appropriate label for this cluster may be “Recent Developments of Psychodrama."

**Table 5:** Citing papers belonging to cluster #1.

**Reference**	**GCS**	**Coverage**
Biancalani et al. (76)	0	12
Mojahed et al. (77)	0	10
Tümlü and Şimşek (78)	0	10
Nieminen (79)	0	8
Purrezaian et al. (80)	1	8
Ron and Yanai (81)	0	8
Testoni et al. (82)	0	7
Beauvais et al. (83)	6	6
Bucuţă et al. (84)	4	6
Sevi et al. (85)	1	6
Orkibi and Feniger-Schaal (25)	31	6
Yaniv (86)	6	6
López-González et al. (28)	0	6
Gonzalez et al. (87)	2	6
Hamidi and Sobhani Tabar (88)	0	6
Kushnir and Orkibi (89)	1	6
Maya and Maraver (90)	3	5
Wang et al. (29)	2	5
Kipper et al. (91)	5	5
Anastasiadis (92)	0	5
Akbiyik et al. (93)	2	5
Logie et al. (94)	11	5

### Cluster #2: Disciplinary Context

The third largest cluster was named “disciplinary context” and consisted of 95 references. The citing papers belonging to this cluster are represented in Table 6. This cluster contains some publications that are included in the book “Psychodrama: Advances in Theory and Practice” edited by Baim et al. (107). As the book is split into two large sections focusing on psychodramatic theory and innovations in practice and research, respectively, this cluster likewise represents more theoretical works as compared to papers representing other clusters. An interesting entry among the other papers is Davelaar et al. (97)'s series of three studies on the construct validity of the Revised Spontaneity Assessment Inventory (SAI-R). First developed by Kipper and Hundal (108) and subsequently revised by Kipper and Shemer (109), the SAI-R is a measure of spontaneity as a core tenet of Moreno's psychodrama, and a means of standardizing spontaneity since the introduction of an action-based spontaneity test by Moreno (110). The SAI was initially developed alongside a Spontaneity Deficit Inventory as two separate continuums rather than opposites of each other (108). To the authors' knowledge, the SAI-R and SDI remain the only psychometric assessments related to psychodrama currently. Taken together, the proposed label for this cluster may be “Theoretical Construction and Measurement of Psychodrama.”

**Table 6:** Citing papers belonging to cluster #2.

**Reference**	**GCS**	**Coverage**
Ruiz-Ruiz (95)	1	15
Schiitzenberger (96)	2	12
Davelaar et al. (97)	7	8
Harkins et al. (98)	14	8
Verhofstadt-Denève (99)	2	8
Meisiek (100)	35	7
Davey et al. (101)	12	6
Miller (102)	1	5
Potik and Schreiber (103)	4	5
Kellermann (104)	2	5
Miller (105)	1	5
Daniel (106)	1	5

### Cluster #3: Psychotherapy

The fourth largest cluster was named “psychotherapy” and consisted of 92 references. The citing papers belonging to this cluster are represented in Table 7, and are the oldest papers when compared to Clusters #0, 1, 2, and 4. In the two papers, there is a focus on action methods, and application of theater to psychotherapy. It can be deduced that this cluster is also a reflection of the growing focus on actionable strategies when implementing psychotherapy, as opposed to the traditional “talk therapy”. Specifically, Chasin et al. (112) described a case study of role-playing various scenarios, both real and fictional, within the context of a couples therapy. The action methods described are similar to those espoused by psychodrama, involving a look into the individual's past issues and role-playing multiple characters in order to gain fresh insight into potential solutions. On the other hand, MacCormack (111) argued that there is a close relationship between elements of theater and psychotherapy, an insight similarly gleaned by Moreno decades ago. Through an integration of theater mechanics and other psychotherapies extending beyond psychodrama and drama therapy, it was argued that the therapeutic process can be made more creative, improvisational and more relatable to the client's social world (111). Again, these are also core principles of psychodrama that show tremendous potential in transforming modern psychotherapy. Taken together, a potential label for this cluster may be “Applications of Psychodrama in Psychotherapy.”

**Table 7:** Citing papers belonging to cluster #3.

**Reference**	**GCS**	**Coverage**
MacCormack (111)	15	34
Chasin et al. (112)	18	22

### Cluster #4: Scene-Based Psychodramatic Family Therapy

The fifth largest cluster was named “scene-based psychodramatic family therapy” and consisted of 89 references. The citing papers belonging to this cluster are represented in Table 8. While the representative label of “scene-based psychodramatic family therapy (SB-PFT)” came from Maya et al. (116)'s and Maya et al. (113)'s papers, all documents in this cluster shared a common feature of employing psychodrama in family or group settings, and seemed to focus on group elements such as collaboration and interpersonal dynamics. Firstly, to elaborate on SB-PFT, it is a form of eclectic therapy bringing together psychodrama, family therapy and group psychotherapy (116). In the initial study, SB-PFT was introduced to adolescents with behavioral issues, alongside their families. Participants cited role-play and mirror techniques as the most helpful in the intervention, while pre- and post-intervention tests showed that participants displayed an increase in emotional intelligence and attachment to their parents. The application of psychodramatic techniques in family therapy was earlier conceived of by Perrott (114), who illustrated examples of how certain techniques can be implemented. In more general group settings, other papers in this cluster focused similarly on adolescents and the youth (117, 118). This cluster may reflect the growing popularity of eclectic therapy in modern clinical psychology, as well as a potential direction of psychodrama in its application in family systems and young people. Taken together, a proposed label for this cluster may be “Psychodrama in Group Contexts.”

**Table 8:** Citing papers belonging to cluster #4.

**Reference**	**GCS**	**Coverage**
Maya et al. (113)	0	15
Perrott (114)	3	13
Frick (115)	1	11
Maya et al. (116)	2	11
Treadwell et al. (117)	7	10
Acquaye et al. (118)	0	9
Acarón (119)	4	8

### Keyword Analysis

The keyword analysis has largely supported the findings of the DCA clusters, although it must be noted that some keywords are general to all scientific publications and not specific to psychodrama (e.g., “article,” “English abstract,” and “methodology”). Nonetheless, it can be observed that the earliest trends in psychodrama research align with the understanding of neuroses at that time, while the later trends reflect a growing rigor in the scientific method through the inclusion of reviews, pre- and post-test design, longitudinal follow-up, as well as qualitative interviews. Additionally, later research trends have broadened in their inclusion of various demographics, such as adults, students, and the aged. The most prominent keywords in the field are intuitively broad in their scope (e.g., “therapy” and “role playing”), although the inclusion of the term “central nervous system” is unexpected, given the lack of research investigating the neurological aspect of psychodrama. This may have to do with the study of neurosis in relation to the central nervous system in older literature.

### Limitations

Despite the relatively robust findings, the study is not without limitations. Firstly, there are limitations to using Scopus as the only database for document search, as Scopus does not have records of all publications. For example, the earliest writings on psychodrama from the 1920s to early 1940s are not found on Scopus. Inclusion of other landmark works from the early days of psychodrama could have changed the final visualised network in significant ways. Additionally, CiteSpace operates on mainly Web of Science bibliographic formatting guidelines and other database records would need to be converted into the Web of Science format prior to analysis. Of the global databases that allow for automatic conversion on CiteSpace, there are three options: Scopus, Web of Science, and PubMed. Therefore, we opted for the database that had the most number of results based on the search terms adopted in order to provide the largest coverage possible of the research field while navigating these constraints. While Scopus produced 2056 records, Web of Science produced 1,788 records and PubMed produced 1,221 records. Future studies may consider making use of other or multiple databases to capture more documents. Alternative databases that may be considered include the aforementioned Web of Science and PubMed that are more compatible with CiteSpace. On the other hand, other databases such as PsycINFO can also be considered in conjunction with other visualization software such as HistCite, Sci2, SciMAT, and VOSViewer. Secondly, depending on the keywords used, the results reflected on any database will vary. While the keyword chosen for this study (“psychodrama*” appearing in either title, abstract, or keywords) is relatively simple and straightforward, relevant articles that did not mention psychodrama in these fields would have been unintentionally excluded from this study. Finally, at a methodological level, there are limitations with the scientometric method itself. This is because strong citation relationships between articles are not indicative of the quality of the papers, as well as the relationship between them [whether the citing paper is in agreement with, or has disproved, the cited paper; Carollo et al. (35)]. The recent Leiden Manifesto (120) published by prominent scientometricians cautions against the misapplication of citation metrics and recommends the use of quantitative evidence to support findings derived from qualitative literature reviews. Therefore, this study has attempted to provide qualitative summaries of prominent research clusters, and recommends an interpretation of DCA metrics obtained in this study within the context of other similar research fields and not against unrelated research domains.

In spite of the limitations described above, this paper holds research value in having conducted the first scientometric analysis of psychodrama research to the best of the authors' knowledge, thereby providing a broad survey of psychodrama and its advances in theory and practice since its inception.

### Recommendations and Future Studies

Based on the discussion summarizing major research developments in the area of psychodrama, we have found a steadily increasing rigor and variety of research methods and topics in this area. Based on these findings, several methodological issues and difficulties as well as potential future developments in this area of research are described below.

We echo Orkibi and Feniger-Schaal (25)'s sentiment that more transparency in data collection, analysis and reporting is needed. Additionally, there are also additional considerations when implementing a psychodrama intervention. Currently, when entering any form of role-play, theoretical conceptualizations of what constitutes a “role” will have an impact on how clients and therapists construe and act out their roles. Whether the theoretical emphasis of the role lies in internal (121) or external (122) processes, or whether the role evolves based on others' feedback and interaction (123), will have a significant impact on the nature of the prompt issued to the individual. For example, instructions from therapists may be given in terms of behaviors that should be exhibited without heed to the internal state of the individual inhabiting the role, or in terms of personality or cognitive factors that are then extrapolated to behaviors up to interpretation by the individual. Unfortunately, prompts or instructions are often undisclosed in many published papers, with the prevailing assumption being that the participants were instructed to role-play as naturally as possible with minimal instructions. While this approach is useful for ethnographic and clinical studies of how psychodrama is typically conducted, it is unhelpful for systematic design of psychodramatic role-play activities in laboratory-based research. Given the increasing focus on systematic study of psychodrama research, the disclosure of all materials, instructions and prompts would be helpful in ensuring consistency and reproducibility of such research. A simple and immediate recommendation to address this issue is to report all details of prompts given to participants during an experimental session, capturing the nature and mode of delivery of these prompts to the individuals. With enough data, future studies may then consider embarking on a meta-analysis or systematic empirical study to understand if these differences indeed produce different behaviors from participants during a psychodrama session, independent of other experimental conditions.

Furthermore, according to Moreno's psychodramatic theory, there are several techniques of role-playing, including role reversal, mirroring, doubling, as well as dyadic and non-dyadic (group) role-plays (12). The different techniques and their associated processes of change have not received much attention in psychodrama research, which has predominantly focused on clinical studies of the efficacy of whole-of-intervention and integrative psychotherapies. The group modality of psychodrama also opens avenues of research into interpersonal dynamics and interaction. Together with the advancement of neuroimaging techniques, one such method that has become popular in studying social interaction is known as synchrony or dyadic synchrony (124), or a quality of mutual responsiveness between two individuals. There have been many approaches to studying synchrony, ranging from the behavioral (125), to affect matching (126, 127), and even to the synchrony of biological signals such as heart rate (128) and brain activation (129). To date, no study has embarked on synchrony studies in the context of psychodrama. The inclusion of alternative physiological measurements such as heart rate or skin conductance may also be considered in the clinical setting as part of the new movement on biofeedback in psychotherapy (130). Additionally, to build upon Yaniv (86)'s work theorizing the relationship between psychodrama and neurocognition, the utilization of neuroimaging tools would prove powerful in providing evidence in this area.

The last methodological issue is that of measuring the experience of psychodrama. As seen in the rising popularity of pre- post-test designs in the evaluation of psychodramatic interventions, the tools of measurement also have to be considered. As with most clinical interventions, therapists rely mainly on client's reported data, qualitative interviews and their own clinical field notes as an indicator of intervention efficacy. Clinically, Carter (131) proposes a role test, which measures the effectiveness of the psychodramatic intervention by how well the client undergoing psychodramatic role-play can function under duress. If quantitative data is preferred, *post-hoc* questionnaires may be used, but there are currently no psychodrama-specific questionnaires that measure psychodramatic experiences that exist to date. Incidentally, several questionnaire studies have been published that measure attitudes toward role-playing and particularly role-playing games (132, 133), but the questionnaire measures used have not been rigorously validated. With the advent of technology, the possibility of using functional neurophysiological tools to obtain real-time data of the progression of a psychodrama session seems higher than ever, and opens up exciting new avenues of clinical research. Complemented with other modes of data collection (such as *post-hoc* questionnaires, or video recordings), data triangulation can be achieved to glean a comprehensive insight into the entirety of the psychodramatic process.

The future therapeutic applications of psychodrama remain bright, particularly when examined in the context of recent breakthroughs in psychotherapeutic technologies and inter-personal communication, as well as growing interest in community-based avenues of mental health support. For example, as is popular in general medicine, telehealth is an increasingly prominent branch of treatment and consultation (134) that can be similarly applied to psychodrama, as seen in Biancalani et al. (76). The incorporation of online tools allow for more frequent points of contact between clinician and client, opening up avenues for ecologically valid interventions out of the clinic and consistent monitoring, although the ethical issues of constant monitoring and online data collection may need further consideration from both clinicians and researchers. Additionally, exciting developments in virtual reality (VR) technology can be combined with psychodramatic techniques to offer more realistic role-playing experiences (135). Such technology has already been implemented in the performing arts to encourage immersive dramatic experiences (136), and holds potential for similar applications in clinical contexts. Finally, the increasing popularity of role-playing games (RPG), from tabletop RPGs such as Dungeons and Dragons to video game RPGs and massively multiplayer online RPGs (MMORPGs), holds potential in its integration with psychodramatic techniques within already established gaming communities. The relationship between RPGs and psychodrama elements such as creativity is explored in Corrêa (137) and may see future work in attempts to integrate elements of RPGs with psychotherapy and psychodrama (138, 139).

## Conclusion

In sum, the present paper set out with three main aims: (1) to survey the major research trends in psychodrama, (2) to provide insight to potential future research directions, and (3) to evaluate psychodramatic research methods. Based on the scientometric visualization and findings, it is evident that psychodrama has had a rich, yet complex, history in research. From the past century of related research, each domain has adopted psychodrama and its related techniques for their own field of application. In order to enable a unifying view of psychodrama, there is a strong need for more transparency in the implementation of psychodramatic programmes that various studies and researchers take. Researchers in psychodrama are also encouraged to embrace advances in methodological tools and technologies, and incorporate them into related research in order to achieve a more comprehensive and rigorous understanding of the processes of psychodrama.

## Data Availability Statement

The original contributions presented in the study are included in the article, further inquiries can be directed to the corresponding author.

## Author Contributions

ML and GE: conceptualization. ML: methodology, investigation, data curation, and writing—original draft preparation. ML and AC: formal analysis and visualization. ML, AC, SC, and GE: writing—review and editing. SC and GE: supervision. GE: funding acquisition. All authors have read and agreed to the published version of the manuscript.

## Funding

This research was supported by grants from the NAP SUG to GE (M4081597 and 2015-2021) and Ministry of Education, Singapore, under its Academic Research Fund Tier 1 (RG55/18) to GE.

## Conflict of Interest

The authors declare that the research was conducted in the absence of any commercial or financial relationships that could be construed as a potential conflict of interest.

## Publisher's Note

All claims expressed in this article are solely those of the authors and do not necessarily represent those of their affiliated organizations, or those of the publisher, the editors and the reviewers. Any product that may be evaluated in this article, or claim that may be made by its manufacturer, is not guaranteed or endorsed by the publisher.
